# The oncogene *Musashi1* encodes novel miRNAs in breast cancer

**DOI:** 10.1038/s41598-023-40666-9

**Published:** 2023-08-22

**Authors:** Liana Lachinani, Mahboobeh Forouzanfar, Kianoush Dormiani, Bahram Mohammad Soltani, Kamran Dolatshahi, Sayyed Mohammadreza Hakimian, Sadat Dokanehiifard, Mohammad Hossein Nasr-Esfahani

**Affiliations:** 1 Department of Animal Biotechnology, Cell Science Research Center, Royan Institute for Biotechnology, ACECR, Isfahan, Iran; 2 Department of Genetics, Faculty of Biological Sciences, Tarbiat Modares University, Tehran, Iran; 3 Department of Medicine, Najafabad Branch, Islamic Azad University, Najafabad, Iran; 4 Ordibehesht Breast Clinic, Isfahan, Iran; 5 Poursina Hakim Digestive Diseases Research Center, Isfahan University of Medical Sciences, Isfahan, Iran; 6 Department of Human Genetics, Sylvester Comprehensive Cancer Center, University of Miami Miller School of Medicine, Biomedical Research Building, Miami, FL USA

**Keywords:** Biological techniques, Cancer, Computational biology and bioinformatics, Biomarkers

## Abstract

RNA-binding protein Musashi1 (*MSI1*) shows an increased expression level in several cancers and has been introduced as a prognostic marker in some malignancies. It is expected that if any miRNA is encoded by this gene, it might have a role in cancer development or could be considered as a prognostic biomarker. Accordingly, in this study, we aimed to find novel miRNA(s) inside the intronic regions of the *MSI1* gene. Here, we report two novel miRNAs within intron 4 of *MSI1* gene, named MSM2 and MSM3, which were selected among several miRNA precursors predicted by bioinformatic studies. For experimental analysis, corresponding precursor miRNAs were transfected into HEK293T cells and exogenous expression of the mature miRNAs were detected. Two mature miRNAs, MSM3-3p and MSM3-5p were generated by MSM3 precursor and one, MSM2-5p was derived from MSM2. Besides, endogenous expression of MSM2-5p and MSM3-3p was detected in MCF-7 and SH-SY5Y cell lines. Expression of both mature miRNAs was also detected in clinical samples of breast cancer. Additionally, the interaction between the MSM3-3p and 3′UTR region of PDE11A was confirmed by dual luciferase assay. Overall, our data demonstrated that *MSI1* gene encodes two novel miRNAs in breast cancer cells.

## Introduction

miRNAs are small non-coding RNA molecules that bind to the 3′UTR regions of target genes to regulate their expression post-transcriptionally. miRNAs play critical roles in many cellular processes and developmental pathways. It is documented that aberrant expression of miRNAs is associated with several disorders. In cancer biology, miRNAs may play the tumor suppressor or oncogenic roles and their expression profile differs in different stages of a particular cancer^1,2^. Also, due to their high stability in different tissues and body fluids, miRNAs are considered as potential non-invasive biomarkers^3,4^. Different stages of cancer, including diagnosis/prognosis, remission, relapse and metastasis have specific miRNA signatures, which contain valuable information applicable for the clinical management of cancer. miRNA signature may be important in determining the drug efficacy in cancer treatment^5^. For the discovery of novel miRNAs, several approaches including experimental and computation-driven methods are employed. In these two methods, miRNAs with a very low expression rate are not detectable. Moreover, non-conserved miRNAs and some miRNAs due to their complex structure and inability to subclone, are missed^6,7^. In order to reduce the possibility of missing novel miRNAs due to the limitations of these methods, in this study we used the combination of both experimental and computation-driven methods to discover novel miRNA(s) inside *MSI1* gene.

The RNA-binding protein Musashi1 (*MSI1*) plays a critical role in normal cell proliferation as well as the differentiation and development of several organs. MSI1 protein is highly expressed in stem cells of different tissues and its aberrant expression is reported in many tumors^8,9^. MSI1 is considered as an activator in tumorigenesis^10–12^ and its increased expression is reported in many cancers such as lung, pancreas, glioma, breast, and colon cancers^13–15^. However, the function and mechanism of action of MSI1 are not fully understood. Kawahara et. al. showed that MSI1 interrupts translation initiation of MSI1 target mRNAs by competing with eIF4G for binding to PABPC1^16^. MSI1 activates the NOTCH and WNT pathways and consequently increases the cell proliferation and keeps the stemness status of cancer cells^17^. Many studies have shown that knockdown of MSI1 reduces cancerous properties including cell proliferation, radioresistance and invasion. MSI1 knockdown in glioblastoma and medulloblastoma cells resulted in reduced self-renewal and survival of the cancer cells^18,19^. Conversely, reducing the expression of MSI1 protein led to increasing the rate of apoptosis and decreasing the cell proliferation in triple-negative breast cancer cell lines by targeting the Notch pathway^20^.

In the present study, we investigated whether the oncogene *MSI1*, which is known as a prognostic factor in several cancers such as breast, ovary, glioma and renal carcinoma^14,21–23^ encodes any miRNAs. Moreover, we tried to determine if there is a possible overlap between the expression pattern or function of the novel miRNAs and their host gene. Using the computational-driven methods, intronic regions of the *MSI1* gene were investigated for potential precursor miRNAs. Two precursor structures were predicted within intron 4 of *MSI1* and among four potential mature miRNAs, two were confirmed experimentally and also detected in clinical samples of breast cancer. Besides, in the clinical samples with low levels of MSI1, we did not detect the expression of the novel miRNAs, which suggests an MSI1-dependent expression regulatory mechanism for these miRNAs.

## Materials and methods

### Bioinformatic prediction of potential precursors and target genes

Several bioinformatic websites were used to predict potential miRNA precursors in the *MSI1* gene. Intronic regions of MSI1 were screened by Sequence, Structure and Conservation profiler (SSCprofiler) tool (http://mirna.imbb.forth.gr/SSCprofiler.html) at both orientations. Input sequences were scanned for stem-loop structures in 1 kb windows with approximately 500 bp overlaps. Then, each of the stem-loop structures was analyzed in MiPred (http://server.malab.cn/MiPred/predict.do), miRNA-dis (http://bioinformatics.hitsz.edu.cn/ miRNA-dis), Fixed-order Markov model (FomMIR) prediction algorithm (http://app.shenwei.me/cgi-bin/FOMmiR.cgi), MiR-Find database (http://140.120.14.132:8080/MicroRNAProject-Web/), and Computational Identification of miRNA (CID-miRNA) web-server (http://mirna.jnu.ac.in/cidmirna/).

In these software, stem-loops were double-checked for the parameters of MFE (Minimum Free Energy), presence of potential Drosha/Dicer cleavage sites and production of mature miRNAs. Stability, MFE and centroid secondary structure of RNA stem-loop structures were studied again by RNAfold program (http://rna.tbi.univie.ac.at/cgi-bin/RNAWebSuite/RNAfold.cgi). Sequences of potential mature miRNAs were blasted in miRBase database (https://www.mirbase.org/) to eliminate the previously discovered analogous miRNAs. The conservation status of candidate structures was examined in UCSC. For each potential miRNA, target genes were predicted by DIANA (http://diana.imis.athena-innovation.gr/DianaTools/index.php?r=mrmicrot/index) and TargetScan (http://www.targetscan.org/vert_50/seedmatch.html) according to their seed regions. Common genes with the highest scores were selected for further experimental analysis. Interaction between predicted miRNAs with 3′UTR regions of candidate target genes was double-checked by RNA22 online software (https://cm.jefferson.edu/rna22/).

### DNA constructions

Specific primers for amplification of miRNA precursors were designed at 100–150 bp of flanking sequences on each side of the target region by Oligo7 (Table 1A). PCR-amplified fragments were subcloned at the *Sal*I and *Xba*I sites of the pBudCE4.1 vector (Cat. No. V53220; Thermo Fisher Scientific Inc.) downstream of the CMV promoter. To track the transfected cells, EGFP reporter was subcloned at *Not*I and *Xho*I sites in EF1α-containing cassette of the vector. For negative control, a universal scramble precursor^24^ was subcloned into the pBud-EGFP vector at *Sal*I and *Xba*I sites (Table 1B).

**Table 1:** List of primers used in this study.

Primer name	Primer sequence (5′–3′)
(A) Primers used for amplification of miRNA precursors	
MSM2 precursor	F: GGATCCTACCCAGGAGTACACCCAGATGC
	R: GTCTAGAGGGACAATCACACCCGCTTTTCC
MSM3 precursor	F: GGATCCAGCTCCTTCGTGTCTTAGACC
	R: GTCTAGACAAATTCTTGGGCACAAGC

Oligomer name	Oligomer sequence (5′–3′)
(B) Universal scramble oligomers	
UScr S	TCGACCCGCTTGTTCGTTGGTAACTACATTCAAGAGATGTAGTTACCAACGAACAAGCTTTTTT
UScr AS	CTAGAAAAAAGCTTGTTCGTTGGTAACTACATCTCTTGAATGTAGTTACCAACGAACAAGCGGG

Primer name	Primer sequence (5′-3′)
(C) Primers used for amplification of wild-type/mutant 3′UTR regions of the target genes	
3′UTR of NCAM1	F: CTCGAGCTCCATTAACATCCCTACC
	R: GCGGCCGCGAACACAAGTATTCCGACAG
3′UTR of mutant NCAM1	F: CATCGATACGTTTCCATTCTCACTGG
	R: GAATGGAAACGTATCGATGTTTCAC
3′UTR of mutant BNC2*	F: GTAAATTCATTTACTGCAGG
	R: CTGAGCCTTGGTCTTCATATCC
3′UTR of mutant PDE11A*	F: GCTGTCTCTGCCTGGAGAAAGTAGAGG
	R: CTACTTTCTCCAGGCAGAGACAGC
3′UTR of mutant ELAVL2*	F: GTGTTTCAGCTACATTTCTTTTC
	R: GAAAAGAAATGTAGCTGAAACAC
3′UTR of mutant PRKAA2*	F: CTGATAGGGCTTTGAACATC
	R: GAAGAGCATACAAGATAACAC

*Wild-type 3′UTR fragments of the specified genes were amplified by the primers listed in Table 2.

For dual luciferase assay, psiCHECK2 (Cat. No. C8021; Promega) reporter vectors containing wild-type or mutant 3′UTR regions of the target genes were constructed. In mutant 3′UTR segments, putative miRNA binding sites were deleted completely by site-directed mutagenesis. Specific primers are listed in Table 1C. 3′UTR fragments were subcloned downstream of Renilla luciferase at *Sac*I and *Sal*I sites and final DNA constructs were verified by sequencing.

### Cell culture and transfection

Cancerous cell lines MCF-7, SKBR3, T47D (breast carcinoma), SH-SY5Y (Neuroblastoma), NTERA2 (testicular teratocarcinoma), LNCaP (prostate carcinoma), and normal breast cell line MCF10A as well as HEK293T cell lines were obtained from the Pasteur Institute of Iran (Tehran, Iran). All cell lines except MCF10A were cultured in DMEM-high glucose (Gibco) supplemented with 10% FBS (Gibco), 1% penicillin/streptomycin (Gibco) and 2 mM L-glutamine (Gibco). MCF10A cells were cultured in DMEM/F12 (Gibco) supplemented with 5% horse serum (Atocel), 10 μg/mL insulin (Sigma), 100 ng/mL cholera toxin (Sigma), 0.5 mg/mL hydrocortisone (Sigma), 20 ng/mL EGF (Sigma), 100 units/mL penicillin and 100 μg/mL streptomycin (Gibco), and 2 mM L-glutamine (Gibco). pBud-EGFP expressing vectors containing miRNA precursors, scramble or wild-type/mutant 3′UTR fragments were transfected into HEK293T cells by Lipofectamine LTX (Cat. No. 15338100; Invitrogen) and 48 h after transfection, the cells were collected for expression analysis or dual-luciferase reporter assay.

### Reverse Transcription Quantitative PCR

For transcription analysis of the target genes and predicted miRNAs, total RNA was extracted by TRI Reagent solution (Cat. No. AM9738; Thermo Fisher Scientific Inc.). For mRNA expression analysis, cDNA was synthesized by RevertAid First Strand cDNA Synthesis Kit (Cat. No. K1621; Thermo Fisher Scientific Inc.) according to the manufacturer’s instruction. Briefly, 1 µg of total RNA was treated by DNaseI (Cat. No. 18047019; Thermo Fisher Scientific Inc.) to get rid of DNA contamination and cDNA synthesis was performed by random hexamer primers. RT-qPCR reactions were performed by 50 ng of cDNA, specific primers (Table 2) and SYBR Premix ExTaq II (Cat. No. RR820Q; TAKARA) in triplicate sets on StepOnePlus Real-Time PCR System (Applied Biosystems). Data of RT-qPCR were normalized using *GAPDH* as the internal reference gene and data analyzed by 2^−∆∆Ct^ or 2^−∆Ct^ methods. In 2^−∆∆Ct ^as a relative method, expression fold changes of the target RNA in the test samples were compared to a single control sample. For different cancer cell lines, which are independent sample cases, we compared expression levels by 2^−∆Ct^. For miRNA expression analysis, cDNA was synthesized from total RNA by miRCURY LNA Universal RT microRNA PCR kit (Cat. No. 203301; Exiqon). MiRNA specific real-time PCR primers were designed by Exiqon technical support team according to predicted mature miRNA sequences. RT-qPCR data were normalized by U6 and analyzed using  2^−∆∆Ct^ method.

**Table 2:** List of the primers used for RT-qPCR.

Primer name	Accession number	Primer sequence (5′–3′)
BNC2	NM_017637.5	F: TCTTCTCCTCTAAAGTGCTGTTG
		R: CTGAGCCTTGGTCTTCATATCC
NCAM1	NM_000615.5	F: CTGGAATGCTGAGTATGAG
		R: GACGAAGATGACGATGAG
PDE11A	NM_001077196.1	F: GTGTAGAACCCTGTCATCAAT
		R: CTGAACAACCTCCTCTTACC
ELAVL2	NM_001385695.1	F: ATTGACGGAATGACCAGTT
		R: TGCCACAGGATACTCTCA
PRKAA2	NM_006252.4	F: GGATTACTGTCATAGGCAT
		R: GAAGAGCATACAAGATAACAC
GAPDH	NM_001357943.2	F: CCACTCCTCCACCTTTGACG
		R: CCACCACCCTGTTGCTGTAG

### Dual-luciferase reporter assay

Direct interaction between predicted novel miRNAs and putative target genes was investigated by the Dual-Luciferase Reporter Assay System kit (Cat. No. E1910; Promega). Wild-type or mutant 3′UTR containing vectors were co-transfected with miRNA-expressing vectors into HEK293T cells by Lipofectamine LTX (Cat. No. 15338100; Invitrogen). After 48 h, Renilla/Firefly luciferase activity was measured at 480 and 560 nm wavelengths, respectively. Renilla luciferase activity in each group was normalized by corresponding firefly luciferase activity.

### Clinical samples

Clinical samples were collected during surgical excision of malignant tumors from breast cancer patients. The patients were enrolled in Ordibehesht Clinic and Askarieh Hospital and their records were used for data collection. For each sample, both malignant and marginal normal tissues were collected and stored immediately in liquid nitrogen. Total RNA was extracted from frozen tissues by TRI reagent solution. Informed consent was obtained from all participants prior to use of the tissues for scientific research. All methods were performed in accordance with the relevant guidelines and regulations. Also, all experiments on human breast normal and cancer tissue samples were reviewed and approved by the ethical committee of the National Institute for Medical Research Development (ethics code: IR.NIMAD.REC.1396.012).

### Statistical analysis

The data was examined using GraphPad Prism 8 (Dotmatics) and presented as mean ± SEM (standard error of the mean). Independent student t-test and ANOVA were applied for analysis to show statistical significance between the groups.

## Results

### Bioinformatic predictions

**Figure 1:**
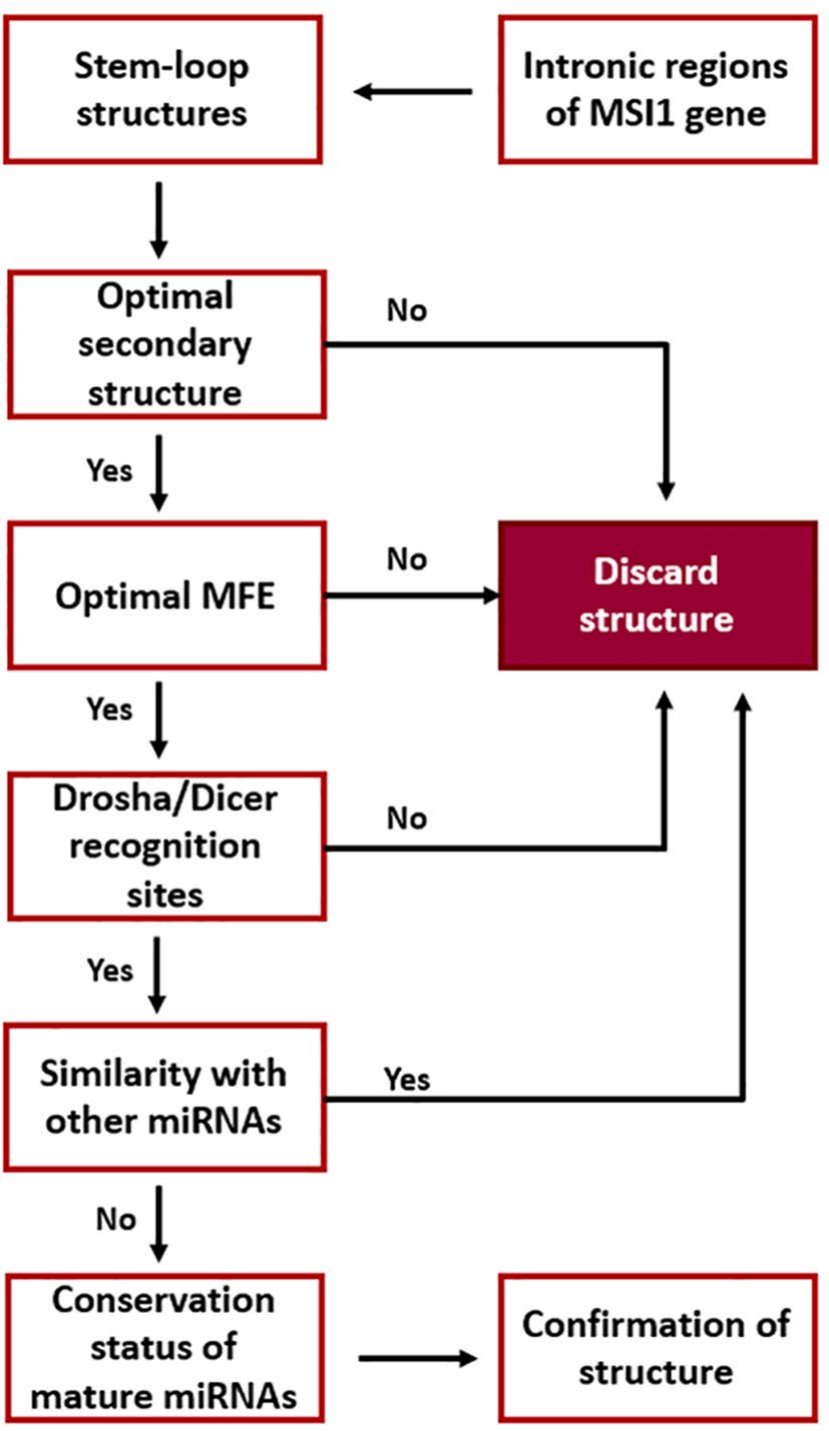
Flowchart of bioinformatic pipeline used for prediction of novel miRNAs. Different software and criteria used in each step have been described in the main text.

**Figure 2:**
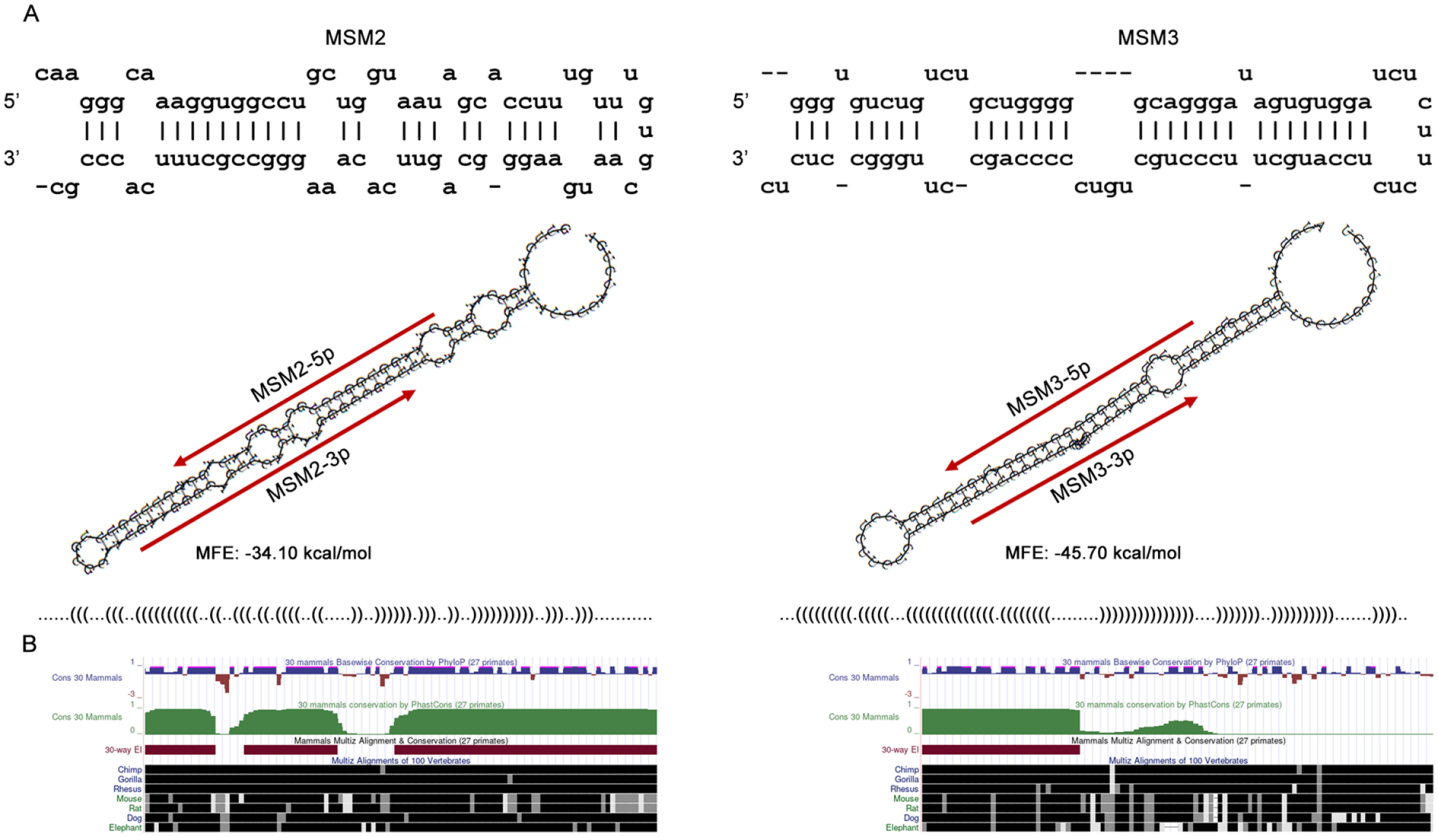
Structural features of predicted precursors. (**A**) Secondary structure and minimal free energy of MSM2 and MSM3 miRNA precursors. Positions of predicted mature miRNAs have shown by red arrows. (**B**) Conservation status of predicted precursors MSM2 and MSM3 according to UCSC (University of California, Santa Cruz genome browser).

In the present study, all 14 intronic regions of the *MSI1* gene were explored to find stem-loop structures. So far, 15 transcript variants have been reported for *MSI1* gene in NCBI and UCSC genome browsers. However, intronic regions of *MSI1* was selected according to the longest transcript variant (transcript variant 1) that contains all intronic regions of the gene. Totally 40 stem-loop structures were detected in both strands of intronic regions. In all structures, several parameters including minimum free energy, Drosha-Dicer recognition sites and conservation were analyzed to explore the most reliable precursor structures. The flowchart of bioinformatic studies is illustrated in Fig. 1. All the structures were detected by the SSCProfiler website, and pre-miRNAs with unusual loops or many branches along with their secondary structure were omitted. In addition to the secondary structure of stem-loops, their free energy was analyzed in RNAfold, and stem-loops with extremely high or low MFEs were removed from the study. The optimal MFE range was assumed between -30 to -55 kcal/mol. To distinguish the pseudo miRNA precursors from the real ones, the structures were analyzed in MiPred and miRNA-dis websites and those that were recognized as pseudo precursors in both websites were omitted. Three websites, FomMIR, MiR-Find and CID-miRNA were used to determine if the selected precursors could produce potential mature miRNAs. Among the predicted 5p/3p mature miRNAs, the mature miRNAs with the highest scores and the same seed regions predicted by different software were selected. In the next step, the conservation of predicted precursors was analysed in UCSC. According to BLAST search programs of MiRBase, none of the candidate mature miRNAs were among the previously reported miRNAs in miRNA databases. Finally, two structures with the highest scores in CID-miRNA and MiPred called MSM2 and MSM3, were selected for experimental studies. Structural features of MSM2 and MSM3 are shown in Fig. 2.

### Predicted mature miRNAs in HEK293T cells

**Figure 3:**
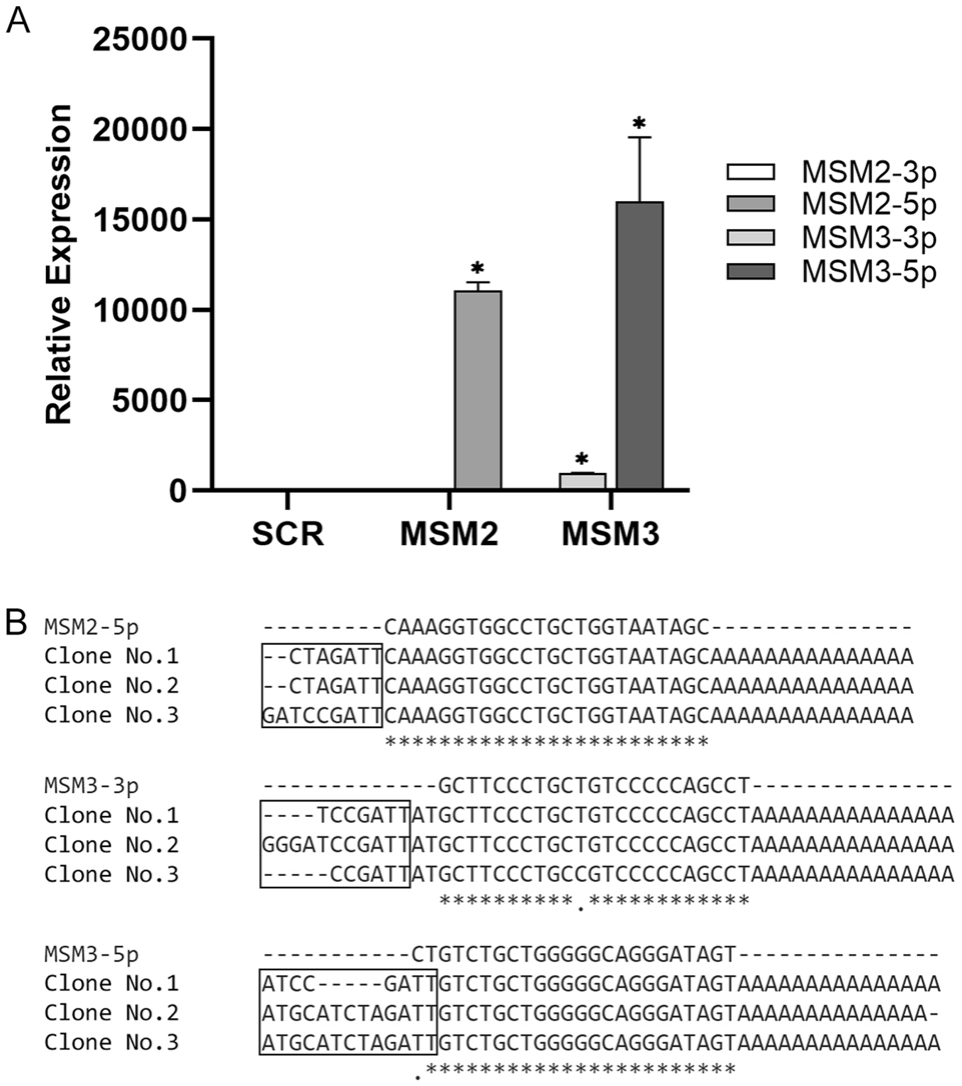
Exogenous expression of predicted miRNAs. Expression and production of mature miRNAs were evaluated in HEK293T cells, which were transfected with miRNA precursors. (**A**) Relative expressions of mature miRNAs from 5′ arm of MSM2, called MSM2-5p and 5′ and 3′ arms of MSM3, called MSM3-5p and MSM3-3p, respectively. Data are represented as the average of triplicates ± SEM, *p-value < 0.05. (**B**) Sequencing results of RT-qPCR products. RT-qPCR products of exogenously expressed miRNAs were subcloned into the pTZ57R/T vector. For each novel miRNA, three random clones were selected for sequencing analysis. The first rows represent the sequences of predicted mature miRNAs and the next rows show the sequences of amplified fragments from different clones. The pTZ57R/T backbone nucleotides are shown in rectangular boxes.

HEK293T cells were transfected by pBud-EGFP-MSM2, pBud-EGFP-MSM3 and scramble vectors separately. After 48 h, the efficiency of transfection was evaluated by fluorescent microscopy and cells were collected for expression analysis. According to RT-qPCR analysis, MSM2 precursor produced only one mature miRNA from its 5′ arm, which was named MSM2-5p. The other mature miRNA from the 3′ arm was not detected while the MSM3 precursor produced both of the predicted mature miRNAs, MSM3-5p and MSM3-3p (Fig. 3A). Sequencing analysis of RT-qPCR products confirmed the generation and expression of three predicted miRNAs (Fig. 3B).

### Detection of endogenous MSM2-5p and MSM3-3p in human cell lines and tissue samples

**Figure 4:**
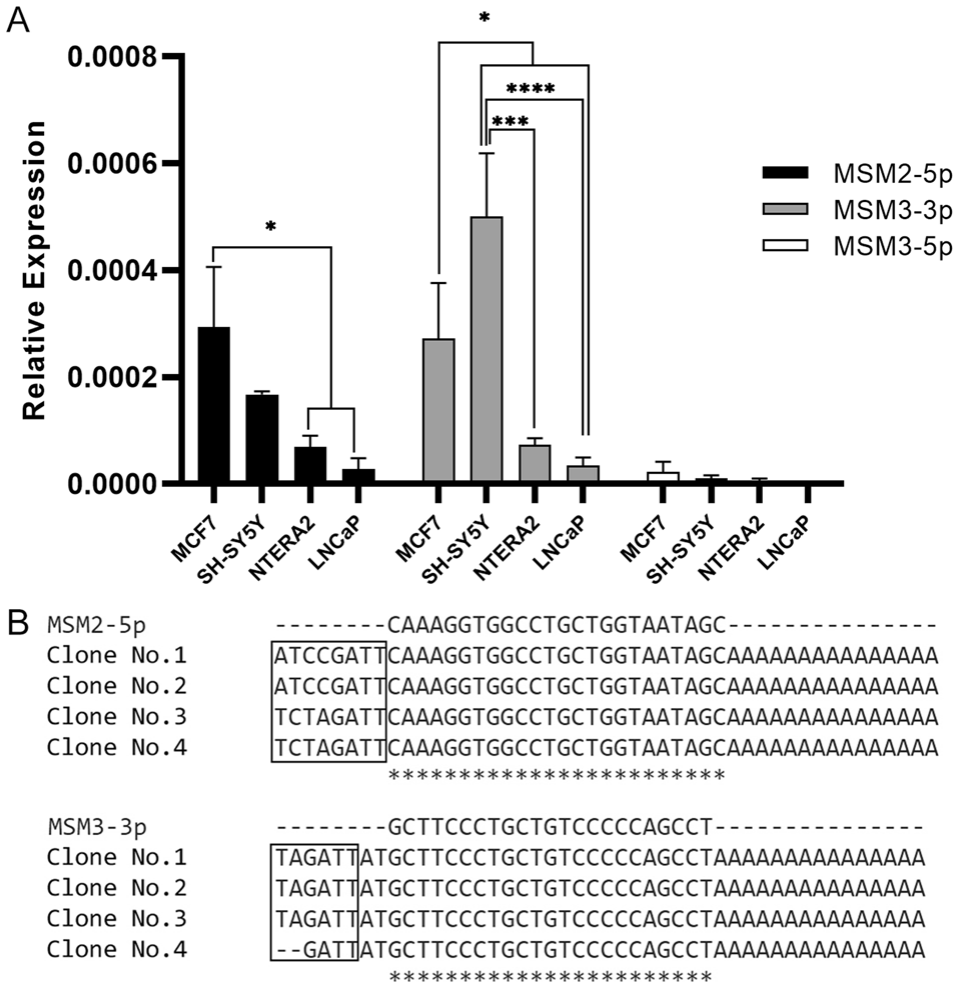
Endogenous expression of novel miRNAs in cancerous cell lines; MCF7, SHSY5Y, NTERA2 and LNCaP. (**A**) Relative expression of MSM2-5p, MSM3-3p and MSM3-5p were calculated using 2^−∆Ct^ method. Error bars denote SEM for three independent repeats in each experiment (*p-value < 0.05, ***p-value < 0.001, ****p-value < 0.0001). (**B**) RT-qPCR products of endogenously expressed miRNAs were subcloned into the pTZ57R/T vector and four random clones were selected for sequencing analysis. The first rows represent the sequences of predicted mature miRNAs and the next rows show the sequences of amplified fragments. The pTZ57R/T backbone nucleotides are shown in rectangular boxes.

To determine the endogenous expression of predicted miRNAs, different cancerous cell lines including NTERA2, SH-SY5Y, MCF-7 and LNCaP were analyzed. Significant expression of two miRNAs, MSM2-5p and MSM3-3p were observed in MCF-7 and SH-SY5Y cells but MSM3-5p expression was not detected in any of the cell lines (Fig. 4A). RT-qPCR products were selected randomly, subcloned into pTZ57R/T vector and analyzed by sequencing. The sequencing results confirmed the endogenous expression of predicted miRNAs (Fig. 4B). As mentioned previously, endogenous expression of MSM3-5p was undetectable and related RT-qPCR products could not be subcloned for sequencing analysis. Thus, we removed this miRNA from the study.

**Figure 5:**
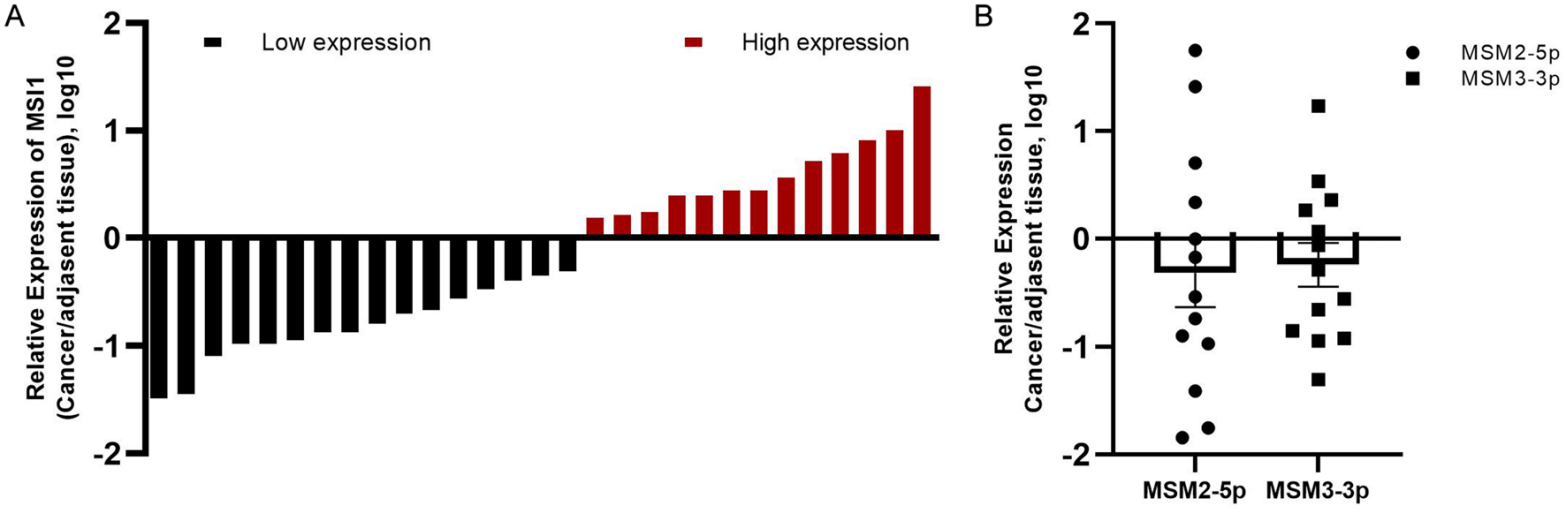
Expression level of MSI1 and MSI1-encoded novel miRNAs in breast cancer samples. (**A**) The mRNA expression level of MSI1 was assessed in 29 pairs of breast cancer and adjacent normal samples. In each cancerous sample, the MSI1 level in cancerous tissues was compared to the corresponding adjacent normal tissues. Data is represented as log10 of cancer to normal tissue ratios. (**B**) MSM2-5p and MSM3-3p expression in clinical samples with increased MSI1 level. Data analysis was performed by 2^−∆Ct^ method and represented as log10 fold changes, n = 13.

To explore the expression of the novel miRNAs in clinical samples, we chose breast cancer samples due to the high expression level of both candidate miRNAs in MCF-7 cells (Fig. 4A). Clinical samples were collected from 29 breast cancer patients. Most of the tumors were assessed as the grade II (55%), stage 1A (24.1%) or 2A (20.6%) and T2 (51.7%). The MSI1 expression level of the cancerous samples was measured in comparison to adjacent normal tissue samples. In most of the cases, MSI1 expression was decreased (Fig. 5A). Expression of the novel miRNAs was also evaluated in clinical samples with increased levels of MSI1. The majority of these tumor samples contained the following parameters: grade II (69.2%), stage 1A or 2A (30.7%) and T2 (61.5%). Although the novel miRNAs showed both decreased and increased expression patterns, they represented a dominantly reduced trend in clinical samples (Fig. 5B).

### Novel MSM3-3p miRNA directly targets PDE11A

**Figure 6:**
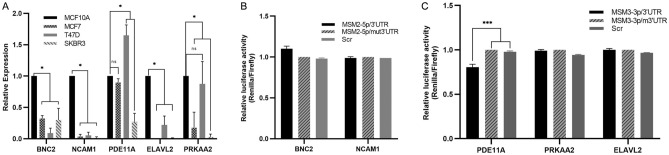
Experimental validation of novel miRNAs. (**A**) Expression level of five predicted target genes was estimated in three breast cancer cell lines in comparison to MCF10A cells as a normal breast cell model. Error bars indicate mean ± SEM for three independent repeats in each experiment, which were considered to be significant at * *p-value* < 0.0001. (**B**) Dual luciferase activity assay was used to determine the direct interaction between predicted miRNA, MSM2 and putative target genes, *BNC2* and *NCAM1* by co-transfection of MSM2 and normal/mutant 3′UTR reporter vectors into HEK293T cells. Each data point represents mean ± SEM for three independent repeats. (**C**) Dual luciferase activity assay was used to determine the direct interaction between predicted miRNA, M3M3 and putative target genes, *PDE11A*, *PRKAA2* and *ELAVL2* by co-transfection of MSM3 and normal/mutant 3′UTR reporter vectors into HEK293T cells. Each data point represents mean ± SEM for three independent repeats, and lines indicate the period of significance at *** *p-value* < 0.001.

For the prediction of potential target genes of novel miRNAs, DIANA and Targetscan websites were used. For each novel miRNA, more than 100 common putative target genes were predicted on both websites. As *MSI1* acts as an oncogene, predicted genes with tumor suppressor function were prioritized in our study. *BNC2* and *NCAM1* were selected as the two most reliable MSM2-5p target genes and *PDE11A*, *ELAVL2* and *PRKAA2* were chosen as the target genes for MSM3-3p. The complementation of predicted target genes and miRNAs was ascertained using RNA22. Due to the high expression level of both novel miRNAs in the MCF-7 cell line, we investigated the expression of potential target genes in MCF-7 and two other breast cancer cell lines T47D and SKBR3. All the predicted target genes had reduced expression patterns at least in one of the cancerous cell lines, which were in favor of selecting them as potential targets for the predicted miRNAs (Fig. 6A).

For experimental validation of the target genes, HEK293T cells were co-transfected with miRNA-expressing and luciferase reporter vectors containing wild-type or mutant 3′UTR regions of the target genes. As shown in Fig. 6B and C, we observed a decreased luciferase activity only in one group, which confirmed a direct interaction between MSM3-3p and the 3′UTR region of *PDE11A*. Thus, the experimental results also confirmed that *PDE11A* could be considered as one of the potential target genes for miRNA MSM3-3p. However, for MSM2-5p we could not detect any direct interaction with the predicted target genes (Fig. 6B).

## Discussion

miRNAs as the most studied small non-coding RNAs, play crucial roles in both normal and irregular biological processes. Most of the miRNA genes are located at the intronic regions of the genome and tend to support their host genes with significantly correlated expression patterns^25,26^. Consequently, finding the novel intronic miRNAs could help to better understand the role of the host genes, especially when the host gene has complicated functions during different biological pathways. *MSI1* is one of the key genes with complicated function and expression patterns in several disorders^8,27^. This gene is well studied in neural stem cells and is known as a neural stem cell marker, however recent studies have also shown that it plays a vital role in many dysregulated pathways that lead to cancerous status^28,29^. Moreover, MSI family members are mentioned as biomarkers in many cancers^21–23^. Searching in miRNA data banks showed that no miRNA encoded by the *MSI1* gene has been reported so far. Accordingly, we decided to determine the presence of novel miRNA(s) inside the gene. There are many approaches to discover novel miRNAs. The traditional method, which is based on cloning of the small RNA fraction is time-consuming and many miRNAs with low expression are undetectable by this method. This problem is solved by the combination of bioinformatic techniques and next-generation sequencing methods^6,7^. Nevertheless, when it comes to finding the novel miRNAs inside a single gene, these techniques are not affordable due to their high outputs. However, the different bioinformatic algorithms they employ could be adjusted for single gene studies. In this regard, by using bioinformatic approaches, we predicted two miRNA precursor structures, which were named MSM2 (GenBank accession number: OQ318158) and MSM3 (GenBank accession number: OQ318159). Both of the precursors are located at the highly conserved regions of intron number 4 in the *MSI1* gene. For experimental confirmation of the predicted precursors, first, we evaluated the ability of pre-miRNAs to generate the mature form of the miRNAs in HEK293T cells. RT-qPCR and sequencing analysis confirmed the production and expression of three predicted miRNAs: MSM2-5p, MSM3-5p and MSM3-3p. One of the miRNAs, MSM2-3p, was not detected even after several PCR optimizations. As commonly reported, in some cases the strand bias may exist such that only the 5′ arm of pre-MSM2 is expressed in HEK293T cells^30,31^, therefore we omitted MSM2-3p from the study.

We also assessed the endogenous expression of predicted miRNAs in several cancerous cell lines. Intronic miRNAs typically show similar expression patterns with their host genes. *MSI1*, as a suggested oncogene and stem cell marker, has shown an increased expression in many cancer types. For example, according to previous studies and Human Protein Atlas, MSI1 exhibits medium/high expression levels in MCF7, NTERA2, LNCaP and SH-SY5Y cell lines. Consequently, we expected the same expression pattern for MSI1-derived miRNAs in these cancerous cell lines. In this study, endogenous expression of MSM2-5p and MSM3-3p were confirmed in tested cancer cell lines. The expression of the miRNAs was more considerable in MCF-7 and SH-SY5Y cell lines so the results encouraged us to select breast cancer as the target malignancy to continue the investigation.

By bioinformatic studies up to 100 target genes were predicted with both Targetscan and DIANA websites for each novel miRNA. As *MSI1* and MSI1-derived miRNAs showed increased expression levels in our study, we omitted the candidate target genes with high expression levels in MCF-7 cells. Then, we removed oncogenes from the list of target genes despite a decrease in their expression level. The screening criteria and scores that we employed for target gene prediction led to a short list of target genes. Moreover, using dual luciferase assay, we confirmed the direct interaction between PDE11A mRNA and MSM3-3p. *PDE11A* encodes a member of the PDEs (Phosphodiesterases) protein superfamily, which modulates a wide range of cellular functions and some studies suggest a tumor suppressor role for PDE11A in adrenal tumors^32,33^. According to D’Andrea et. al., expression of PDE11A was detected at an equal levels in both normal and cancerous breast tissues by immunolabeling^34^. In our experiments, we observed three different expression patterns for PDE11A in three distinct breast cancer cell lines. In T47D and SKBR3 cells, *PDE11A* showed up and down-regulation patterns, respectively but no significant differences were observed in MCF-7 in comparison to normal MCF10A cells. Despite the same context of estrogen/progesterone receptor (ER/PR) status of MCF-7 and T47D, the results demonstrated that the expression of PDE11A was not the same. Proteomic analysis of these cell lines has shown that the expression level of the proteins that are involved in cancerogenesis, cell growth and anti-apoptosis mechanisms is higher in T47D cells^35^.

For investigation of the novel miRNAs expression in clinical samples, we collected the clinical samples from breast cancer patients according to the high expression levels of MSI1 in MCF-7 cells. Breast cancer is a highly heterogeneous disease and several types of this cancer show different characteristics and complex gene expression patterns. Typically, among the different types of breast cancer, the expression of MSI1 is increased significantly in estrogen/progesterone (ER/PR) positive cells^14^. But among the collected clinical samples, this classification was not confirmed such that many ER/PR positive samples showed a decrease in expression of MSI1. It seems that ER/PR positivity does not guarantee the high levels of MSI1 in the clinical samples. Therefore, the expression assessment of the predicted miRNAs was restricted to the clinical samples with increased MSI1 irrespective of their marker receptor status.

As a result, both up and down-regulation patterns of the miRNAs were observed among the samples. We investigated the pathological properties of these two sample groups to find an association between the cancer type and miRNAs expression pattern but we could not find any logical association. Regardless of our first plan, we hypothesized a conceivable negative correlation between the *MSI1* as the host gene and its encoded miRNAs. Hence, we also assessed the relative expression of miRNAs in samples with low MSI1 that showed no detectable miRNAs. This finding makes the prospect that novel miRNAs (MSM2 and MSM3) do not transcribe from independent promoters and their expression is regulated in parallel with *MSI1* as their host gene. Exploring the intron 4 of *MSI1 *by GeneHancer database at UCSC genome browser, revealed that both MSM2/MSM3 pre-miRNAs are surrounded by several enhancer-like regions, which may play a significant roles in both the expression regulation of MSM2/MSM3 pre-miRNA and the positive or negative correlation of these precursors with their host gene. To clarify the roles of the novel miRNAs, a larger clinical sample population with more pathological data is required. Previous studies have shown that miRNA profile could change drastically in different stages of a particular cancer type^5,36^. Therefore, it is recommended that the stage and aggressiveness status of the clinical samples are considered in the future studies because it seems that these factors could be the reasons for alteration in the expression patterns that we detected in our study.

In conclusion, our RT-qPCR and sequencing results confirmed the presence of two novel miRNAs inside the intronic region of the *MSI1* gene. The expression of novel miRNAs was confirmed in a number of various cell lines and also in clinical samples of breast cancer. Furthermore, direct interaction between MSM3-3p and *PDE11A* as a tumor suppressor gene suggests the oncogenic role for the miRNA. To resolve the variable expression patterns of predicted miRNAs observed in clinical samples, it is also recommended that a large number of breast cancer patients are included in the study by attention to the details of pathological findings in each group.

## Data Availability

The datasets generated during the current study are available in the GenBank repository, MSM2; OQ318158; https://www.ncbi.nlm.nih.gov/nuccore/OQ318158.1, and MSM3; OQ318159; https://www.ncbi.nlm.nih.gov/nuccore/OQ318159.1.
